# Assessing cross-species transmission of hemoplasmas at the wild-domestic felid interface in Chile using genetic and landscape variables analysis

**DOI:** 10.1038/s41598-019-53184-4

**Published:** 2019-11-14

**Authors:** I. Sacristán, F. Acuña, E. Aguilar, S. García, M. J. López, A. Cevidanes, J. Cabello, E. Hidalgo-Hermoso, W. E. Johnson, E. Poulin, J. Millán, C. Napolitano

**Affiliations:** 1 PhD Program in Conservation Medicine, Facultad de Ciencias de la Vida, Universidad Andres Bello, República 252, Santiago, Chile; 2 Facultad de Ciencias Veterinarias y Pecuarias, Universidad de Chile, Avda. Santa Rosa 11735, La Pintana, Santiago Chile; 3 Facultad de Medicina Veterinaria, Universidad San Sebastián, sede de la Patagonia, Puerto Montt, Chile; 4 Centro de Conservación de la Biodiversidad Chiloé Silvestre, Ancud, Chile; 5 Departamento de Conservación e Investigación, Parque Zoológico Buin Zoo, Panamericana Sur Km 32, Buin, Chile; 6 Walter Reed Biosystematics Unit, Smithsonian Institution, 4210 Silver Hill Rd., Suitland, MD 20746 USA; 7 Smithsonian Conservation Biology Institute, National Zoological Park, 1500 Remount Road, Front Royal, VA 22630 USA; 8 Instituto de Ecología y Biodiversidad (IEB), Las Palmeras 3425, Ñuñoa, Santiago Chile; 9 Laboratorio de Ecología Molecular, Departamento de Ciencias Ecológicas, Facultad de Ciencias, Universidad de Chile. Las Palmeras 3425, Ñuñoa, Santiago Chile; 10 Facultad de Ciencias de la Vida, Universidad Andres Bello, República 252, Santiago, Chile; 11 Departamento de Ciencias Biológicas y Biodiversidad, Universidad de Los Lagos, Av. Fuchslocher 1305, Osorno, Chile

**Keywords:** Infectious-disease epidemiology, Bacterial infection

## Abstract

The co-occurrence of domestic cats (*Felis silvestris catus*) and wild felids in rural landscapes can facilitate pathogen transmission. However, in the relatively-isolated regions of southern South America there have been no comprehensive studies to assess disease transmission risks between domestic cats and forest-dwelling wild felids such as guigna (*Leopardus guigna*). We evaluated hemoplasma infection and the possibility of transmission between domestic cats and guignas by comparing spatial and phylogenetic patterns of pathogen prevalence. Blood/spleen samples were collected from 102 wild guignas and 262 co-occurring rural domestic cats across the entire distribution range of guigna in Chile. Hemoplasma infection was assessed by direct sequencing of the 16S RNA gene. Infection with hemoplasmas was common and geographically widespread across different bioclimatic areas for both species. The most common feline *Mycoplasma* species in guigna and domestic cats were *Candidatus* M. haemominutum (*C*Mhm) (15.7% guigna; 10.3% domestic cat) and *Mycoplasma haemofelis* (*Mhf*) (9.8% guigna, 6.1% domestic cat). A previously undescribed *Mycoplasma* sp. sequence was found in two guignas and one cat. Continuous forest-landscapes were associated with higher hemoplasma*-*prevalence in guignas. Shared hemoplasma nucleotide sequence types between guigna and domestic cats were rare, suggesting that cross-species transmission between guignas and domestic cats may occur, but is probably uncommon. Ectoparasites, which have been linked with hemoplasma transmission, were not found on guignas and were infrequent on domestic cats. Our results suggest that transmission pathways vary among hemoplasma species and, contrary to our predictions, domestic cats did not appear to be the main driver of hemoplasma infection in guignas in these human-dominated landscapes.

## Introduction

Anthropogenic landscape change has been identified to be one of the main drivers of pathogen emergence in wildlife^1^, because of the increased opportunities for cross-species pathogen transmission among domestic animals and wildlife in human-dominated landscapes^2^. Phylogenetic relatedness, interspecific interactions and geographic overlap among host and vector species are main predictors of pathogen spillover^3^. Domestic cats and wild felids, which share a common ancestor a relatively recent 10 million-years ago^4^, often share genetic, physiological and ecological traits and thus share similar susceptibility to many infectious agents^2^. These shared biological features and the ubiquitous presence of domestic cats around human settlements make co-occurring domestic and wild felids an important study system to investigate frequency and impact of pathogen spillover at the wildlife-human interface^5^. Transmission of pathogens from domestic to wild felids has been linked with population declines among wild species, such as the case in bobcats (*Lynx rufus*) with the outbreak of feline panleukopenia virus in 1988^6^, and the outbreaks of feline leukemia virus (FeLV) in Iberian lynx (*Lynx pardinus*) in 2006^7^ and Florida panther (*Puma concolor coryi*) in 2001–2004^8^ and 2010–2016^9^. Therefore, gaining a better understanding of the mechanisms, ubiquity, and patterns of pathogen transmission among domestic and wild felids has epidemiological, ecological and conservation implications.

Haemotropic mycoplasmas (aka hemoplasmas), formerly classified as *Hemobartonella* and now identified as *Mycoplasma* spp., are obligate epierythrocytic bacteria that parasite red blood cells^10^. Mycoplasmas present an absence of cell walls and small genomes, making them strictly dependent on the host cell. In contrast with several mucosal hemoplasmas, hemoplasmas have never been grown successfully in culture^11^. Hemoplasmas infect a wide range of mammals and are distributed worldwide^12^. Three hemoplasma species are known to infect felids: *Mycoplasma haemofelis* (*Mhf*), *Candidatus* Mycoplasma haemominutum (*C*Mhm) and *Candidatus* Mycoplasma turicensis (*C*Mt)^13–15^. Prevalence of feline hemoplasma infection ranges from 10 to 40% in domestic cats worldwide^11,16–20^. In general, *C*Mhm is the most prevalent species in domestic cats, followed by *Mhf* and *C*Mt^12,17,20,21^. *Mhf* is also the most pathogenic and can cause hemolytic anemia in immunocompetent cats. Hemoplasma acute infection can induce hemolytic anemia, anorexia, lethargy, dehydration, weight loss and sudden death^17^. The chronic form has been associated with the absence of clinical signs. More studies to elucidate the long-term implications of hemoplasma infection are necessary^11^.

In wild felines, hemoplasma infection with *C*Mhm, *Mhf* and/or *C*Mt has been reported worldwide, including in tigers (*Panthera tigris*), Iberian lynx, leopard cat (*P*. *b*. *euptilurus*), European wildcat (*Felis silvestris silvestris*), several Brazilian felid species, Iriomote cat (*Prionailurus bengalensis iriomotensis*) and African lion (*Panthera leo*)^22–28^. Hemoplasma sequences from wildlife species often share close identity to those found in domestic cats^23,25,27^. Based on genetic evidence, domestic cats may be the source of the global distribution of multiple strains of hemoplasmas in wild felids. Persistent onward transmission following spillover may be possible due to high prevalence among some species of wild felids^23,27^.

The epidemiology and transmission patterns of hemoplasmas are poorly understood^12^. Geographical aggregation of infection in some studies relates transmission with an arthropod vector^29^, and the cat flea, *Ctenocephalides felis*, has been proposed to be a potential vector in *Mhf*^30^. However, other experimental studies have found no evidence of hemoplasma transmission by fleas^31^. Alternatively, cat fights could also be a route of hemoplasma transmission since subcutaneous inoculation of *C*Mt infected blood in laboratory settings resulted in transmission, whereas saliva did not^32^. If this were common, then transmission by aggressive interaction would be more likely than transmission by social contact (saliva via mutual grooming)^32^. However, for *C*Mhm (but not for *Mhf*), there is evidence of horizontal transmission by direct contact between cats but no observed signs of aggressive interaction or vectors^29^. Additionally, the discovery of the feline hemoplasma species, *C*Mt, which is closely related to rodent hemoplasmas, supports the hypothesis of predator-prey transmission of these agents between mice and cats^33^. Finally, it has been suggested that blood transfusions might be a potential route of transmission^34^.

Here we assess the prevalence and cross-species pathogen transmission among small felids in rural landscapes of central and southern Chile. In these areas non- feral free-roaming domestic cats are abundant and can roam up to 2 km from their human households during incursions into native forest habitat (unpublished GPS tracking devices and camera trap data). This roaming behavior increases the probability of contact with forest-dwelling wildlife species and other domestic animals and thus increases the chances of coming in contact with a range of pathogens. Moreover, these domestic cats are rarely subjected to any prophylactic programs or veterinary care, increasing their possibility of being infected with pathogens^35^. To date, there have been no systematic studies or records documenting the existence of completely feral cat populations in Chile.

The forest-dwelling guigna (*Leopardus guigna*) is a small wild felid inhabiting central and southern Chile (30°–48°S) and a narrow strip of land in southwestern Argentina (39°-46°S west of 70°W)^34^. It is one of the most threatened wild felids in the Americas and is classified by the IUCN as Vulnerable with declining populations^36^. Guignas are solitary and closely associated with native forests, as they depend on vegetation cover^37–39^ and the small mammals and birds prey species. However, they also inhabit small forest patches surrounded by a matrix of human-modified habitats (i.e., livestock and agriculture) where domestic cats are often present^40^, providing opportunities for contact that may facilitate pathogen spillover. Moreover, guignas in these human-dominated landscapes have disperse greater distances and occupy larger ranges, rising the chances of contact with other species. These populations also have reduced genetic diversity, making them potentially more susceptible to infectious diseases^41^. Possible cross-species transmission from domestic cats to guignas of FeLV and feline immunodeficiency virus (FIV) has been recorded in human-perturbed landscapes in southern Chile^42^. However, there is no information about other pathogens that could be infecting guignas.

In Chile, hemoplasma has been detected in domestic cats in the city of Valdivia (southern Chile) with infection prevalence rates between 1–8%^43^ depending on the bacterial species. Similarly the Darwin’s fox (*Lycalopex fulvipes*), a small forest-dwelling canid endemic to southern Chile, has a high infection prevalence of *Mycoplasma* spp. (57% of 30 individuals)^44^. Although they are found throughout the current distribution of Darwin’s foxes and coexist with rural domestic cats in human-perturbed landscapes, hemoplasma infection has never been described in guignas.

Here, we used epidemiology, phylogenetics and landscape ecology to understand patterns of hemoplasma variation at the wildlife-domestic interface. We carried out an extensive molecular survey for hemoplasma infection in guigna across its entire distribution range in Chile and in co-occurring rural domestic cats. We evaluated hemoplasma infection and prevalence and modelled possible transmission pathways and landscape drivers associated with infection. We conducted genetic analyses based on sequences of the bacterial 16S rRNA gene to identify hemoplasma species infecting several known host species and evaluate the existence of shared genotypes as evidence of cross-species transmission. To understand the possible impact of hemoplasma infection on guigna health, we also assessed the clinical status of hemoplasma-infected guignas.

We hypothesized that human-dominated landscapes provided increased opportunities for domestic cat-guigna encounters and cross-species pathogen transmission. Therefore, we expected to find i) higher hemoplasma prevalence in guignas inhabiting human-dominated landscapes compared with pristine continuous forest habitats, and ii) high genetic similarity (and shared genotypes) among domestic cat and guigna hemoplasma sequences.

## Materials and Methods

### Study area

**Figure 1:**
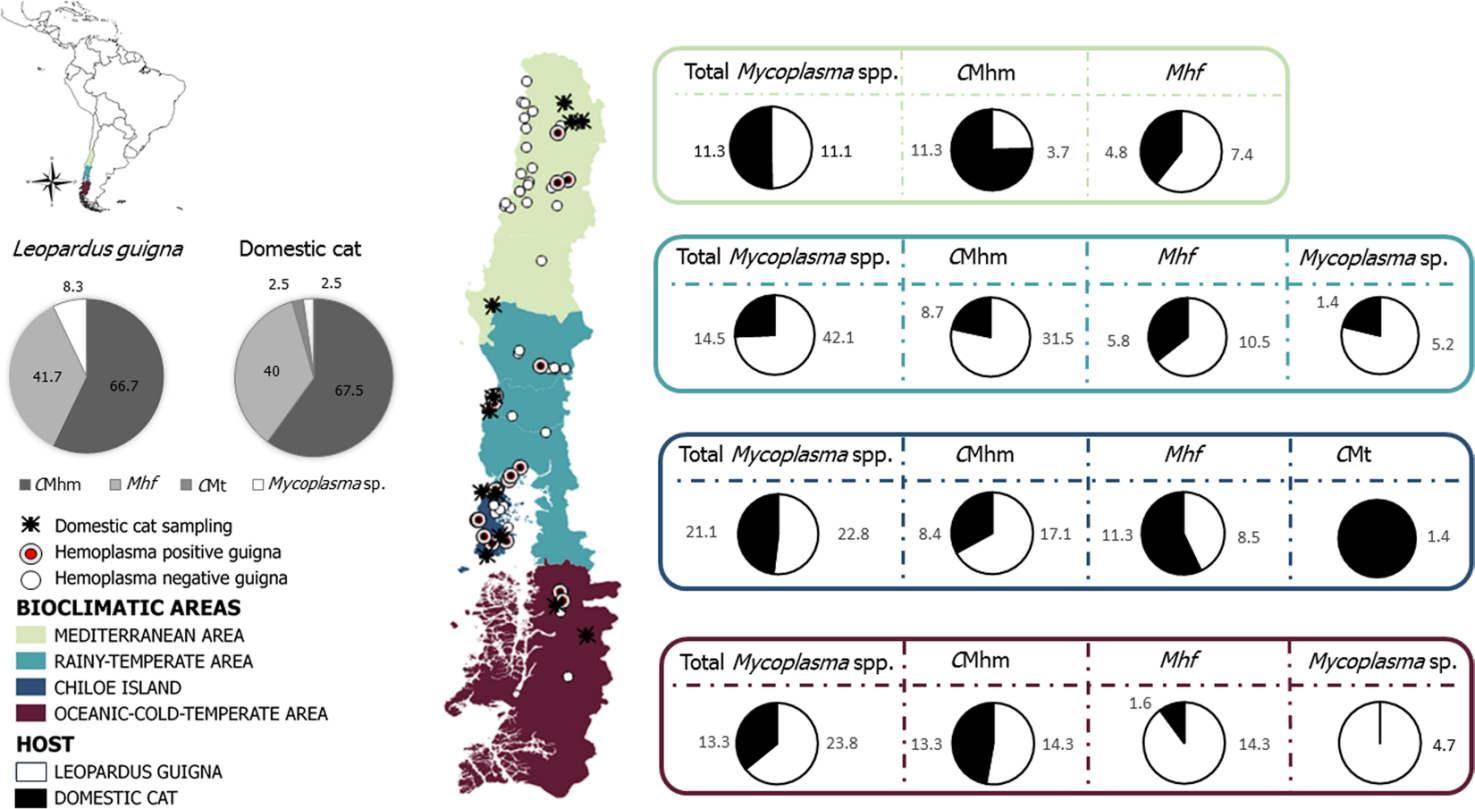
Map of study area, sampling sites and prevalence of each hemoplasma species (including coinfections) in guignas and domestic cats (left grey scale pie charts) and across bioclimatic areas (guigna in white, domestic cat in black). Mhf= Mycoplasma haemofelis, CMhm= Candidatus Mycoplasma haemominutum, CMt= Candidatus Mycoplasma turicensis.

The study was conducted across the entire current distribution of guignas in Chile (33°S–46°S)^36^, encompassing four different bioclimatic regions (Fig. 1). The study sites included a gradient of different landscape types, ranging from continuous pristine native forest with no human presence to human-perturbed landscapes with high human densities and where fragments of remnant forest are surrounded by a matrix of agricultural, livestock along with their domestic pets (cats and dogs) and other human activities.

### Sample collection

From 2008 to 2018, 102 free-ranging guignas were sampled after being captured in tomahawk-like live traps baited with chicken and olfactory attractor (Hawbakers wildcat lure; Fort Loudon, PA, USA) (n = 52) or after being received injured in wildlife rescue and rehabilitation centers (WRRC; n = 8). Whole blood samples were collected from these 60 animals by cephalic or jugular venipuncture in EDTA tubes. In addition, 42 spleen samples were collected during complete necropsies of “road-killed” cats found dead or following euthanasia at WRRC. Guigna captures and tissue collection were conducted using standard protocols^41^ that conformed with handling and supervision protocols within bioethical and animal welfare frameworks^45^ and with permission from the Chilean Agriculture and Livestock Service (SAG) (capture permits 814/13 2008, 109/9 2009, 1220/22 2010, 1708/26 2010, 7624/2015, 2288/2016, 2185/2017, 4072/2018). The anesthesia protocol (5 mg/kg ketamine and 0.05 mg/kg dexmedetomidine) was adapted from methods described in other wild felid species from South America as *L*. *colocolo*^46^. For each guigna we recorded the GPS location of collection/capture, sex, age class (estimated from dentition patterns), physical condition, season and presence of ectoparasites. Any clinical signs of disease were assessed and recorded by a veterinarian. Of 38 sampled females and 64 males, 63 were adults and 16 juveniles (no age data was available for 23 of the animals).

We sampled 262 free-roaming domestic cats from rural communities across the entire distribution of guigna in Chile. Whole blood samples (n = 254) were collected by cephalic or jugular venipuncture using manual restraint after the owner’s consent. Eight spleen samples were collected during complete necropsies of domestic cats found as road-kills or euthanized at veterinary clinics. The sex, age class, season and “capture” location of each cat was recorded. The presence of ectoparasites was assessed in 125 domestic cats (48%). In total, 129 females and 133 males, 226 adults and 36 juveniles were sampled.

All procedures were conducted with full consideration of animal welfare and ethical protocols and with the approval of Animal Ethics Committee of the Institute of Ecology and Biodiversity in Universidad de Chile (resolution of 20 November 2015). Samples were stored frozen at −20 °C until needed for molecular analyses.

### Genetic analysis of hemoplasma sequences

Total DNA from 100 µl of blood and 10 mg of spleen was extracted using a DNeasy® Blood & Tissue Kit (Qiagen, Germany) following the manufacturer’s instructions. To monitor for cross-contamination during the extraction process, negative controls consisting of 100 µl phosphate-saline buffer were concurrently prepared with each batch of 15 samples. We used universal primers^44^ to amplify 391 bp (base pairs) of the 16S rRNA gene from a broad range of hemoplasma species. PCR conditions consisted in 20 µl volume containing 2.5 µL 10x PCR Buffer, 2.0 µL MgCl2 50 mM, 2.5 µL dNTP, 0.3 U of Platinum Taq DNA polymerase, and 1.0 µl of each forward and reverse primers 10 pmol/µl). Thermocycling parameters consisted on an initial denaturation at 95 °C for 5 min, 40 cycles of 94 °C for 30 seconds, 58 °C for 30 seconds, 72 °C for 30 seconds, and a final extension of 72 °C for 7 min. All samples that were determined to be positive were molecularly characterized by amplifying the complete 16S rRNA gene using a semi nested PCR that amplified 492 bp and 1107 bp of the 16S rRNA gene, with an overlapping fragment of 171 bp to facilitate sequence assembly. The first round conditions were the same for the two PCRs, using Hemo-F1 and Hemo-R2 published primers and PCR conditions^47^, and were subsequently amplified through two different reactions using Hemo-F1/Hemo-R1 primers, and Hemo F2/Hemo-R2 primers^47^ (Table 1).

**Table 1:** Primers used in the present study.

Organism	Primer name	Sense	Sequence 5′-3′	Size (pb)	PCR type	Reference
*Mycoplasma* sp.	Mycop16S rRNA-F	Forward	ATGTTGCTTAATTCGATAATACACGAAA	384	Single	Cabello *et al*.^44^
	Mycop16S rRNA-R	Reverse	ACRGGATTACTAGTGATTCCAACTTCAA			
	HemoF1	Forward	AGAGTTTGATCCTGGCTCAG	492	Semi-nested 2 A	Harasawa *et al*.^47^
	HemoR1	Reverse	ACCGCAGCTGCTGGCACATA			
	HemoF2	Forward	ATATTCCTACGGGAAGCAGC	1107	Semi-nested 2B	Harasawa *et al*.^47^
	HemoR2	Reverse	TACCTTGTTACGACTTAACT			
*Mycoplasma haemofelis*	OH-OK	Forward	ATGCCCCTCTGTGGGGGATAGCCG	273	Single	Watanabe *et al*.^48^
	00CR-r1	Reverse	ATGGTATTGCTCCATCAGACTTTCG			
*Candidatus* Mycoplasma haemominutum	CA-B2	Forward	CTGGGAAACTAGAGCTTCGCGAGC	202	Single	Watanabe *et al*.^48^
	00CR-r1	Reverse	ATGGTATTGCTCCATCAGACTTTCG			
*Candidatus* Mycoplasma turicensis	CMt-F	Forward	AGAGGCGAAGGCGAAAACT	138	Single	Peters *et al*.^21^
	CMt-R	Reverse	CTACAACGCCGAAACACAAA			

To assess the possibility of coinfection by different hemoplasma species, we screened positive samples using species-specific PCR primers that targeted the three recognized species of feline hemoplasma. To detect *Mhf-* and *C*Mhm-derived DNA, one-step PCR amplification was performed following published PCR conditions^21,48^ and using two forward and one reverse oligonucleotide, including a OH-OK *Mhf-*specific forward primer, a CA-B2 *C*Mhm-specific forward primer, and a 00CR-r1 common reverse primer^48^ (Table 1). The CMt PCR amplification used primers CMt-F and CMt-R^21^.

For all PCR assays, ultrapure water was used as a negative control. PCR products were visualized in 2% agarose electrophoresis gel stained with GelRed fluorescent dye. After PCR, all products were purified and sequenced in both directions at Macrogen (South Korea) with primers described above. All sequences were aligned using CLUSTALW algorithm (Geneious®) and compared to those of the GenBank database to assess their uniqueness.

The best model of evolution among sequences was determined with the program jModelTest2 (version 2.1.6)^49^, under Akaike Information Criterion (AIC)^50^ (both for each hemoplasma species separately and altogether) to identify the level of complexity of the model of nucleotide substitution that best fit the combined dataset. Phylogenetic trees were constructed based on Neighbor-Joining, Maximum likelihood and Bayesian methods using GTR + I + G model. Maximum-likelihood analysis was conducted using RaXML software version 1.5^51^. The data set was resampled 1,000 times to generate bootstrap values. We used software iTOL for visualization^52^. Bayesian inference analysis was performed with Mr. Bayes 3.1.2^53^. Four Markov chain Monte Carlo (MCMC) simulations were run for 10^9^ generations with a sampling frequency of every 1000 generations and a burn-in of 25%. Mixing and convergence of the MCMC was assessed through the average standard deviation of the split frequencies (ASDSF), being the optimum closest to zero. Estimated sample size (ESS) values were assess for stabilized variance, targeting values greater than 100. Genetic divergence between clades was calculated as genetic distance (Ks) with DnaSP.5 software^54^.

A nucleotide sequence type (ntST) network was generated using the median joining approach method implemented in popart software (Population Analysis with Reticulate Trees)^55^. Phylogeographic structure of *C*Mhm and *Mhf* in guignas and domestic cats was assessed comparing frequency-based G_ST_ with the pairwise difference-based N_ST_ indices^56^ implemented in Permut^57,58^. The level of significance was assessed with 1000 permutations. Genetic structure of *C*Mhm and *Mhf* in guigna and domestic cat host species was estimated using pairwise Phi_st_ tests implemented in Arlequin^59^ (level of significance assessed with 1000 permutations) and the nearest-neighbor statistic S_nn_^60^ implemented in DnaSP.5^54^

### Nucleotide sequence accession numbers

Newly identified hemoplasma 16S rRNA sequences were submitted to the GenBank database under accession numbers: *C*Mhm A1 (MN543623); *C*Mhm A2 (MN543624); *C*Mhm A3 (MN543625); *C*Mhm A4 (MN543626); *C*Mhm A5 (MN543627); *C*Mhm A6 (MN543628); *C*Mhm A7 (MN543629); *C*Mhm A8 (MN543630); *C*Mhm A9 (MN543631); *C*Mhm A10 (MN543632); *Mhf* B1 (MN543633); *Mhf* B2 (MN543634); *Mhf* B3 (MN543635); *Mhf* B4 (MN543636); *Mycoplasma* sp. C1 (MN543637); *C*Mt D1 (MN543638).

### Hematology

Hematological parameters were evaluated in EDTA blood samples from 19 guignas (11 hemoplasma-infected, 8 hemoplasma-negative) using the Abacus Junior Vet Hematology Analyzer (Diatron®). Biochemical parameters were obtained from 18 guigna serum samples (10 hemoplasma-infected and 8 hemoplasma-negative) and analyzed by Microlab 100 de MERCK® employing Wiener® Lab reactives. Blood smears from 19 sampled guignas were performed. Differential leukocyte count, cell morphology evaluation and hemoparasite searches were performed on Giemsa-stained blood smears using an optic microscope (Carl Zeiss®, modelo Standard 20).

### Landscape analysis

To identify and describe landscape features associated with hemoplasma infection in guigna, surrounding each guigna sample location we generated a circular buffer using the program QuantumGIS 2.14®, that corresponded to the mean home range described for guignas (males = 446 ha; females = 170 ha)^37,61,62^. Within each buffer area, we described and quantified seven landscape variables of land use and human occupation, biologically relevant to assess our questions: (1) percent vegetation cover^63^, (2) presence of houses, 3) number of houses, (4) distance of guigna capture location to the nearest house, (5) land use (fragmented landscape or continuous forest), (6) local administrative region and (7) bioclimatic region (Mediterranean region, rainy temperate region, Chiloe Island: rainy temperate to oceanic cold temperate transition region and oceanic cold temperate region)^64^. GIS layers were obtained from the Ministerio de Bienes Nacionales website^65^ and QGIS 2.14® software was used to extract the landscape attributes.

We tested the collinearity among predictors by running bivariate Pearson correlations among pairs of variables. No statistically significant correlations were found.

To address spatial autocorrelation in our data, we conducted a Global Moran’s I test using ArcGIS Pro. We obtained non-significant results (Moran’s index = 0.38, z-score = 0.46, *p*-value = 0.64) suggesting there is no pattern of data spatial clustering.

### Statistical analyses

Spatial (landscape use) and biological (host age and sex) independent variables and hemoplasma infection (binary response variable) were assessed with multivariate logistic regression analysis (function glm), calculating crude and adjusted odds ratios (ORs) (function confint) within 95% confidence intervals (CIs). Goodness of fit models were assessed using the Hosmer Lemeshow test (function hoslem.test) and an analysis of residuals.

Differences in hemoplasma spp. infection prevalence between domestic cat and guigna, as well as between seasons, were analyzed by Mann-Whitney U tests (function wilcox.test). Hematological and biochemical parameters of hemoplasma-infected and non-infected guignas were compared by Kruskal-Wallis tests (function kurskall.test). Comparisons between flea-infected and hemoplasma-infected domestic cats were conducted using Fisher exact tests. All statistical analyses were performed in R software^66^ with a significance level of *p* < 0.05.

## Results

### Hemoplasma prevalence and genetic diversity

Across the study area, 24 guignas (23.53%; 95% CI = 15.16–31.90%) and 40 domestic cats (15.27%; 95% CI = 10.8–19.65%) were positive for hemoplasma. There was no statistically significant difference observed in prevalence rate between the two species (*p* = 0.28, *U* = 10690). Among the positive cases, 16 guignas (15.7%, 95% CI = 8.5–22.86%) and 27 domestic cats (10.3%, 95% CI = 6.6–14.01%) had *C*Mhm, based on both matches with GenBank sequences (showing 99% similarity to *C*Mhm) (Table 2) and by species-specific PCR. Ten different ntSTs corresponding to *C*Mhm were identified (Table 3), four of which were found solely in guigna (Table 3) and six only in domestic cats. None of the ntSTs were observed in both species.

**Table 2:** Summary of *Mycoplasma* sequences detected in sampled guigna and domestic cats identified by 16S rRNA similarity and BLAST search of NCBI database.

Animal species	Nucleotide sequence type (ntST)	Gene 16S rRNA length (bp) n = n° of sequences	Percentage of identity by BLAST® analysis
Guigna	***C*Mhm A4**	391 bp n = 1	*Candidatus* Mycoplasma haemominutum isolate 56/09B, Domestic cat from Italy, KR905457.1 99%
	***C*Mhm A5**	1289 pb n = 1	*Candidatus* Mycoplasma haemominutum isolate H4 Domestic cat from Brazil, KM275257.1 99%
	***C*Mhm A7**	391 bp n = 1	*Candidatus* Mycoplasma haemominutum isolate H4 Domestic cat from Brazil, KM275257.1 99%
	***C*Mhm A8**	391 bp n = 5 1289 pb n = 8	*Candidatus* Mycoplasma haemominutum isolate Israel no.1 Domestic cat from Israel, AY150974.1 99%
	***Mhf*** **B1**	391 bp n = 1	*Mycoplasma haemofelis* isolate YNKM1 Domestic cat from China, MH447082.1 100%
	***Mhf*** **B3**	391 bp n = 2 1289 bp n = 2	*Mycoplasma haemofelis* isolate YNKM1 Domestic cat from China, MH447082.1 99%
	***Mhf*** **B4**	1289 bp n = 1	*Mycoplasma haemofelis* isolate 574 Eurasian lynx from Switzerland, DQ825458.1 99%
	***Mycoplasma*** **sp C1**	1289 bp n = 2	Uncultured *Mycoplasma* *Lycalopex fulvipes* from Chile, MK457366 99%
Domestic cat	***C*Mhm A1**	1289 bp n = 5 391 bp n = 2	*Candidatus* Mycoplasma haemominutum isolate Purdure 1 Domestic cat from USA, FJ004275.199%
	***C*Mhm A2**	1289 bp n = 1	*Candidatus* Mycoplasma haemominutum isolate 56/09B, Domestic cat from Italy, KR905457.1 99%
	***C*Mhm A3**	1289 bp n = 10 391 bp n = 5	*Candidatus* Mycoplasma haemominutum isolate Birmingham 1 Domestic cat from UK, HE613254.1 99%
	***C*Mhm A6**	1289 bp n = 1	*Candidatus* Mycoplasma haemominutum strain ITI26_2 Domestic cat from Italy, EU839980.1 99%
	***C*Mhm A9**	1289 bp n = 1	*Candidatus* Mycoplasma haemominutum isolate Israel no.1 Domestic cat from Israel, AY150974.1 99%
	**CMhm A10**	391 bp n = 1	*Candidatus* Mycoplasma haemominutum isolate Cat3 Domestic cat from Iran, KU852585.1 99%
	***Mhf*** **B1**	1289 bp n = 1 391 bp n = 10	*Mycoplasma haemofelis* isolate YNKM1, Domestic cat from China, MH447082.1 100%
	***Mhf*** **B2**	1289 bp n = 1	*Mycoplasma haemofelis* from cat 1089 Domestic cat from Switzerland, DQ157156.1 100%
	***Mycoplasma*** **sp**. **C1**	1289 bp n = 1	Uncultured *Mycoplasma* *Lycalopex fulvipes* from Chile, MK457366 100% Uncultured *Mycoplasma* Rodent from Brazil, KT215636.1 99%
	***C*Mt D1**	1289 bp n = 1	*Candidatus* Mycoplasma turicensis isolate D7 Domestic cat from South Africa, DQ464424.1 100%

**Table 3:** Shared and private nucleotide sequence types (ntST) of *Mycoplasma* spp. and number of guignas and domestic cats presenting each ntST (1249 bp 16S rRNA gene).

Hemoplasma spp.	*C*Mhm										*Mhf*				*Mycoplasma* sp.	*C*Mt
NtST name	A1	A2	A3	A4#	A5	A6	A7#	A8	A9	A10#	B1	B2	B3	B4	C1	D1
Guigna n*	0	0	0	1	1	0	1	13	0	0	1	0	4	1	2	0
Domestic cat n*	7	1	15	0	0	1	0	0	1	1	11	1	0	0	1	1

n* = number of individuals with each ntST.

# = ntST identified based only on the 391 bp amplicon of the 16S rRNA gene.

DNA samples from 10 guignas (9.8%, 95% CI = 3.9–15.67%) and 16 domestic cats (6.1%, 95% CI = 3.1–9.0%) were identified as *Mhf*, both through comparison with sequences deposited in GenBank (showing 99% similarity to *Mhf*) (Table 2) and by species-specific PCR. Four different ntSTs were identified, one found solely in domestic cats, two only in guignas, and one that was documented in guignas and domestic cats (Table 3).

A unique *Mycoplasma* sp. sequence was obtained from two guignas (1.9%, 95% CI = 0.7–4.6%) and one domestic cat (0.38%, 95% CI = 0.3–1.1%) (Table 3). Comparison with sequences deposited in GenBank showed 100% similarity to a *Mycoplasma* sequence obtained from a Darwin’s fox in Chile, and 99% similarity with an uncultured *Mycoplasma* sp. from a Brazilian rodent (Table 2). One sequence from a domestic cat (Table 3) had 100% similarity with *C*Mt sequences from GenBank (Table 2).

Results of species-specific PCR revealed coinfection of *C*Mhm and *Mhf* in three guignas (2.9%; 95% CI = 0.3–6.2%) and five domestic cats (1.9%, 95% CI = 0.2–3.5). Coinfection of *Mhf* and *Mycoplasma* sp. was found in one guigna (0.98%, 95% CI = 0.9–2.9%). No coinfections involving *C*Mt were observed.

**Figure 2:**
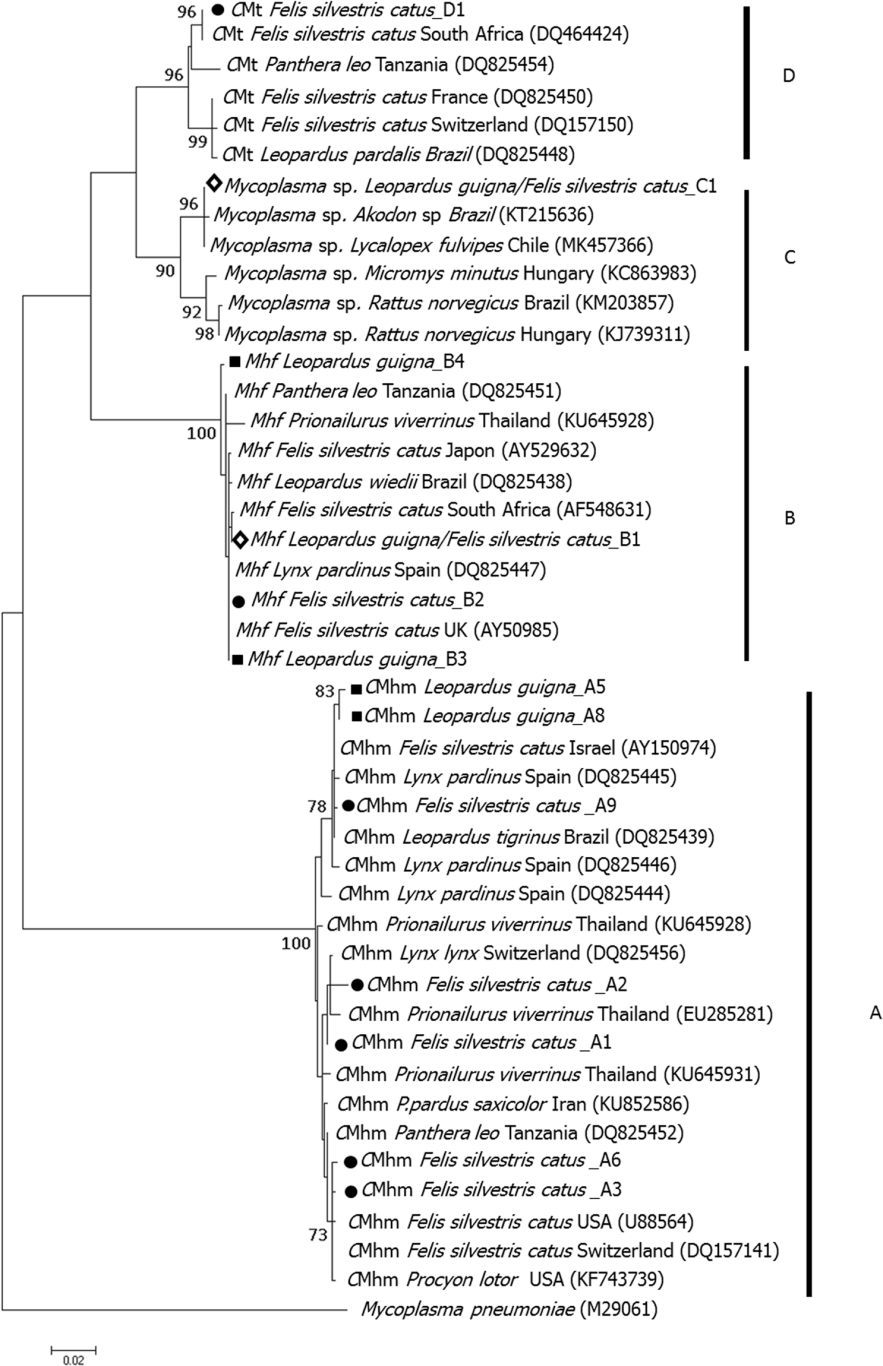
Maximum likelihood tree of 944 bp of the 16S rRNA gene for guigna and domestic cat. *M*. *pneumniae* sequence has been used as outgroup. Bootstrap values of ≥70 are printed at the nodes of the tree. Circles, squares and diamonds mark domestic cat ntST, guigna ntST and shared ntST from our study. The Bayesian phylogenetic tree was congruent. The four phylogenetic (taxonomic) groups are labelled (**A**–**D**).

Molecular characterization of 16S rRNA gene (1289 bp) was obtained for 11 of the 24 hemoplasma-positive guignas and 22 of 40 hemoplasma-positive domestic cats. Each hemoplasma species showed a different evolution model. Neighbor joining trees were constructed to test consistency with Maximum likelihood and Bayesian trees. Full consistency among trees was obtained. Phylogenetic analyses revealed four well-supported clades (A, B, C, D) that corresponded with the three identified hemoplasma species and an unidentified *Mycoplasma* sp. group or OTU (*operational taxonomic unit*) (Figs 2 and S1). The ntSTs from our sampled guignas and domestic cats from Chile were positioned within clades shared with domestic cats, wild felids and other carnivore species from around the world. However, a separate group consisting of two guigna sequences (A5, A8) were observed within the *C*Mhm clade. Genetic distance between clades C and D is lower (0.037) than between clades A and C (0.067), and clades A and B (0.086).

**Figure 3:**
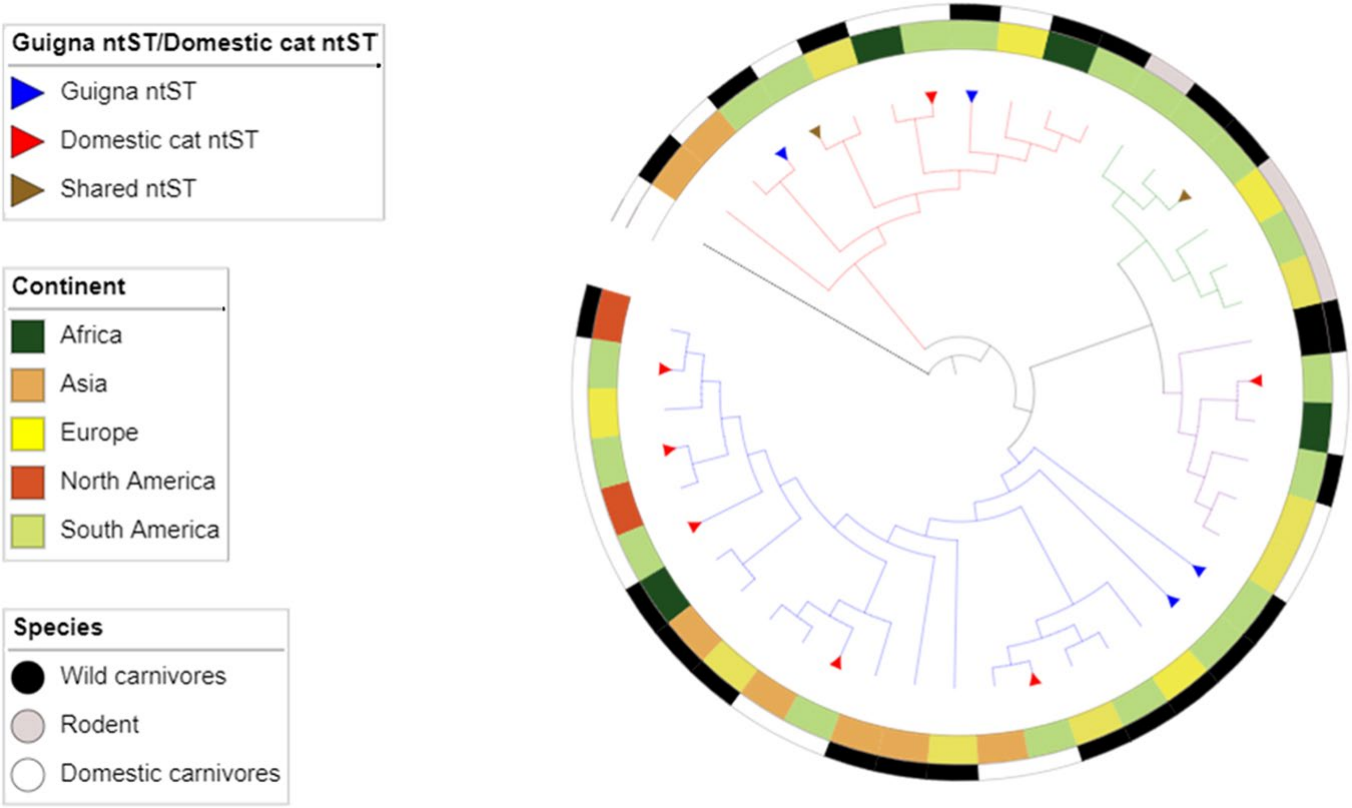
Phylogenetic representation for geographic origin and host species of the hemoplasma sequences reported from this study and worldwide sequences published in GenBank. Maximum likelihood phylogenetic inference of 944 bp of the 16S rRNA gene, constructed using same phylogenetic tree analysis from Fig. 2, same node supports.

Genetic analysis of the most prevalent bacterial species (*C*Mhm and *Mhf*) described here along with world-wide hemoplasma sequences obtained from GenBank revealed no geographic structure (all non-significant paired Phi_st_ tests) or genetic structure among domestic and wild carnivore host species (*C*Mhm: S_nn_ = 0.55, *p* = 0.25; Phi_st_ = 0.07, *p* = 0.15; *Mhf*: S_nn_ = 0.5, *p* = 0.2; Phi_st_ = 0.006, *p* = 0.45) (Fig. 3). Gst-Nst test could not be conducted due to low number of sequences per location.

**Figure 4:**
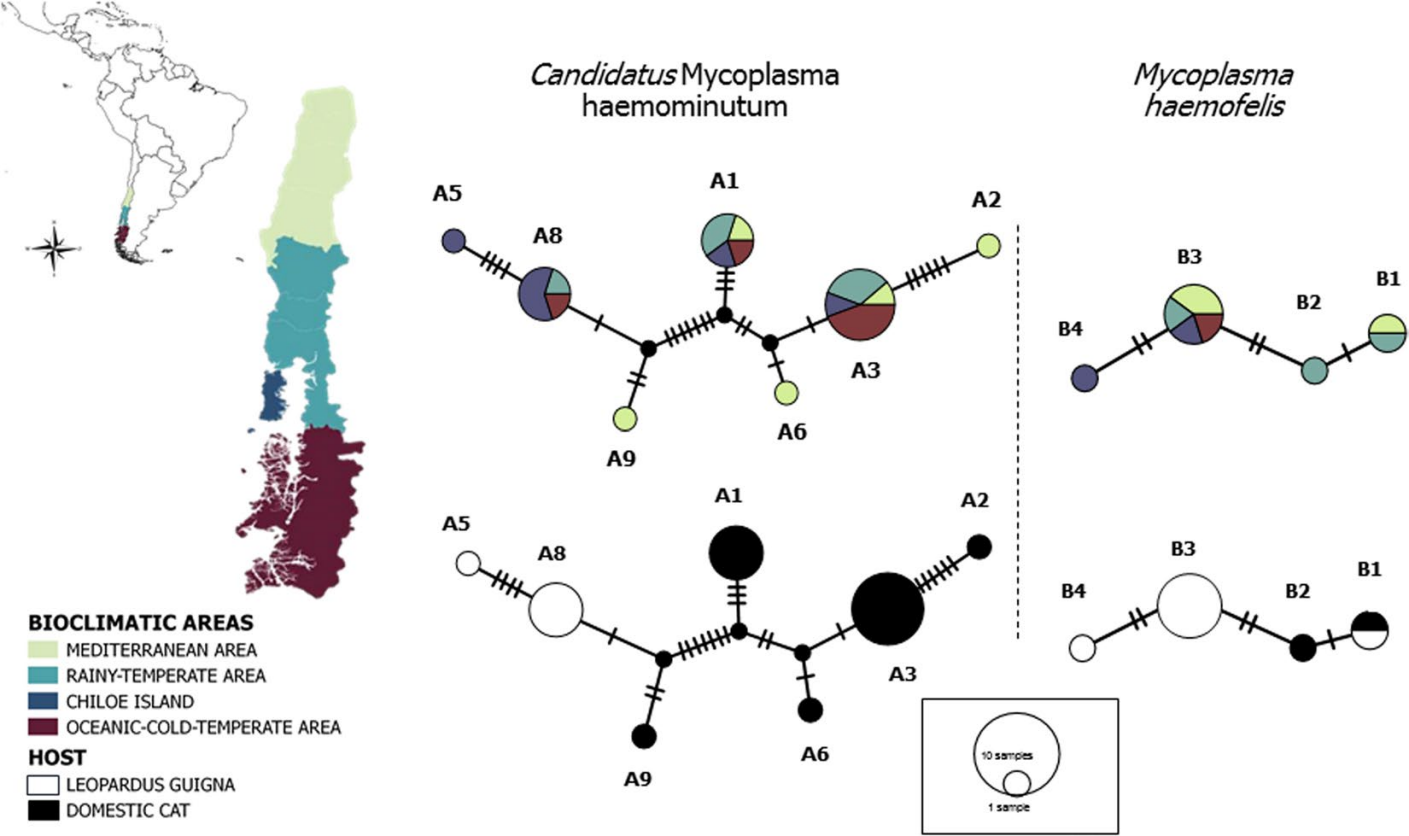
Nucleotide sequence type (ntST) network of guigna and domestic cat *Mycoplasma* sequences (1,289 bp). Each circle in the network corresponds to a different haplotype, the size of the circles correspond to haplotype frequencies, the color of the circles correspond to the four different bioclimatic areas and two host species (*L*. *guigna* and domestic cat).

NtST network analysis (1289 bp) of this study’s *C*Mhm and *Mhf* sequences visually revealed no geographic clustering, as the different ntSTs were widespread throughout the study area (Fig. 4). No phylogeographic structure was found testing Gst-Nst statistics. For both *C*Mhm and *Mhf*, N_ST_ coefficients were higher than G_ST_ values but with non-significant differences (*C*Mhm: N_ST_ = 0.101 ± 0.11, G_ST_ = 0.037 ± 0, *p* = 0.3; *Mhf*: N_ST_ = −0.099 ± 0.04, G_ST_ = −0.019 ± 0.1, *p* = 0.9).

In contrast, genetic structure based on host species for *C*Mhm was visually apparent, as all ntST were species-specific, either unique to domestic cats or unique to guignas (Fig. 4). Statistically significant genetic structure of *C*Mhm sequences between guignas and domestic cats was found, based on contrasting ntST frequencies (Phi_st_ = 0.65, *p* = 0.00) and nucleotide sequence-based statistics (S_nn_ = 0.95, *p* = 0.00). On the other hand, two cases of shared ntSTs between both host species were observed (1 for *Mhf*, 1 for *Mycoplasma* sp.) and no genetic structure of *Mhf* sequences between guignas and domestic cats was found (Phi_st_ = 0.19, *p* = 0.39; S_nn_ = 0.58, *p* = 0.43).

### Exposure risk factor analysis

Although hemoplasma-infected guignas were found in all four bioclimatic areas sampled in Chile (Fig. 1, Table 4), multivariate logistic regression analysis revealed a significantly higher prevalence of total hemoplasma species and for *C*Mhm specifically (Tables 4 and S1, Fig. S2) in the rainy temperate area. We also found a significantly higher prevalence of total hemoplasma species and specifically of *C*Mhm and *Mhf* (Table S1, Fig. S2) in guignas from continuous forest compared with fragmented landscapes. However, there were no differences in infection prevalence between sampling seasons, males and females or among different age classes in guignas. In domestic cats, higher prevalence for total hemoplasma species was observed in males compared to females (*p* = 0.05) but without significant differences. There were no differences between sampling seasons, age classes or bioclimatic areas (Tables S1 and S2).

**Table 4:** Prevalence of hemoplasma species identified in guignas and domestic cats in each bioclimatic area, gender, age, landscape and season.

Hemoplasma species	Host species	Bioclimatic region (Prevalence% 95%CI)				Gender (Prevalence% 95%CI)		Age ** (Prevalence% 95%CI)		Landscape (Prevalence% 95%CI)		Season ** (Prevalence% 95%CI)	
		Mediterranean area	Rainy temperate area	Chiloe Island	Oceanic-cold temperate area	Male	Female	Adult	Juvenile	Fragmented landscape	Continuous forest	Cold season (Winter- Autumn)	Warm season (Spring- Summer)
Sample size (n)	**Guigna**	27	19	35	21	64	38	63	16	69	33	34	66
	**Domestic cat**	60	62	69	71	133	129	226	36	N/A	N/A	108	105
Total *Mycoplasma* spp.	**Guigna**	11.1 1.5–23.7	42.1 17.6–66.5	22.8 8.2–37.4	23.8 3.9–43.6	27.0 15.9–38.9	18.8 5.5–31.0	22.0 12.0–33.0	38.0 11.0–64.0	15.94 7.0–24.8	39.4 21.8–57.0	29.4 13.2–45.5	21.21 11.9–31.3
	**Domestic cat**	11.3 3.1–19.3	14.5 5.9–23.0	21.1 11.4–30.8	13.3 4.4–22.1	19.5 12.7–26.4	10.8 5.4–16.2	16.4 11.5–21.2	8.3 −0.1–17.8	N/A	N/A	13.9 7.2–20.52	10.5 4.5–16.4
*C*Mhm	**Guigna**	3.7 3.7–11.3	31.5 8.5–54.6	17.1 4.0–30.1	14.3 2.0–30.6	18.75 8.9–28.5	10.5 0.3–20.7	14.29 5.4–23.1	18.7 2.7–40.2	10.14 2.8–17–4	27.3 11.2–43.3	17.6 4.1–31.1	15.15 6.2–24.0
	**Domestic cat**	11.3 3.1–19.4	8.7 1.8–15.5	8.4 1.8–15.0	13.3 4.4–22.1	13.5 7.6–19.4	7.0 2.5–11.4	11.1 6.9–15.1	5.6 −2.3–13.4	N/A	N/A	12.0 5.8–18.3	10.5 4.5–16.4
*Mhf*	**Guigna**	7.4 3.1–17.9	10.5 4.6–25.7	8.5 1.1–18.3	14.3 2.0–30.6	10.94 3.0–18.8	7.9 1.1–16.9	9.5 2.1–17.0	25.0 1.2–49.0	4.3 −0.5–9.2	3.0 −3.1–9.2	11.76 0.3–23.18	9.0 1.9–16.21
	**Domestic cat**	4.8 0.0–10.3	5.8 0.0–11.4	11.3 0.3–18.8	1.6 1.6–5.0	7.5 2.3–12.0	4.7 0.1–8.3	6.6 3.3–9.9	2.8 −2.8–8.4	N/A	N/A	3.7 0.8–7.3	3.8 0.8–7.5
*Mycoplasma* sp.	**Guigna**	0.0 0.0–0.0	5.2 5.7–16.3	0.0 0.0–0.0	4.7 5.1–14.7	1.5 −1.5–4.7	2.6 −2.7–8.0	1.5 −1.5–4.8	6.2 −7.0–19.6	1.4 −1.4–4.3	3.0 −0.3–9.2	2.9 –3.0–8.9	1.5 –1.5–4.5
	**Domestic cat**	0.0 0.0–0.0	1.4 1.4–4.3	0.0 0.0–0.0	0.0 0.0–0.0	0.7 −0.7–2.2	0.0 0.0–0.0	0.4 −0.4–1.3	0.0 0.0–0.0	N/A	N/A	0.9 −0.9–2.7	0.0 0.0–0.0
*C*Mt	**Guigna**	0.0 0.0–0.0	0.0 0.0–0.0	0.0 0.0–0.0	0.0 0.0–0.0	0.0 0.0–0.0	0.0 0.0–0.0	0.0 0.0–0.0	0.0 0.0–0.0	0.0 0.0–0.0	0.0 0.0–0.0	0.0 0.0–0.0	0.0 0.0–0.0
	**Domestic cat**	0.0 0.0–0.0	0.0 0.0–0.0	1.4 1.4–4.3	0.0 0.0–0.0	0.0 0.0–0.0	0.7 −0.7–2.3	0.4 −0.4–1.3	0.0 0.0–0.0	N/A	N/A	0.0 0.0–0.0	0.0 0.0–0.0

^*^N/A, not applicable.

^**^No age data was available for 23 guignas, no season data was available for two guignas and 49 domestic cats.

Ectoparasites were found in seven of 125 evaluated domestic cats (5.6%; 95% CI = 1.5–9.6). All ectoparasites were fleas and no ticks were found. Two hemoplasma-positive domestic cats had fleas (1 *C*Mhm, 1 *Mhf*). There was no statistical difference in hemoplasma infection prevalence between flea-positive and negative animals (Fisher exact test; *p* = 0.3). The seven flea-positive domestic cats were found in both cold and warm seasons. We were unable to compare ectoparasite prevalence between bioclimatic zones due to the small number of flea-positive animals. No ectoparasites were found on the guignas.

### Clinical signs in guigna

No clinical signs of hemoplasma infection were observed in any guigna through direct inspection. However, in one *C*Mhm-infected guigna and one *C*Mhm-*Mhf*-coinfected guigna, inclusions in red blood cells compatible with hemoplasma organisms were observed in blood smears (Table S3, Fig. S3). Hematological parameters were obtained from 11 hemoplasma-infected and 8 hemoplasma-negative guignas, while biochemical parameters were obtained from 10 hemoplasma-infected and 8 hemoplasma-negative guignas (Tables S3 and S4). Comparisons revealed no statistically significant differences in hematological and biochemical values between hemoplasma infected and non-infected guignas, although sample numbers for these comparisons were low. Comparison of hematological and biochemical values of hemoplasma-infected guignas with the baseline data from the closely-related *Leopardus geoffroyi*^67^ suggested that two juvenile male guignas had nonregenerative anemia. Both of these individuals were infected by *Mhf* and one was coinfected with *C*Mhm. Both also presented abnormal ALT, albumin and AST values, but neither presented inclusions in red blood cells observed in blood smears. Asymptomatic animals infected by *C*Mhm, *Mhf*, or *Mycoplasma* sp. and coinfected by *C*Mhm and *Mhf* and *Mycoplasma* sp. and *Mhf* were also found.

## Discussion

To our knowledge, this study represents the first large-scale molecular survey of hemoplasma for any co-occurring domestic/wild carnivores. Our remarkably large sample size of a threatened, elusive wild felid such as the guigna, including samples from its entire distribution in Chile, was matched with a large sample of rural domestic cats from the same areas. Genetic analyses revealed that infection with genetically diverse hemoplasmas is common and geographically widespread in both species.

The hemoplasma prevalence of 24% in guigna was within the range described for other species of solitary wild felids^22–26^ and wild carnivores worldwide^28^. The highest prevalence reported for a solitary carnivore was for the Darwin’s fox in southern Chile^41^ (57%). Relatively higher prevalences have been observed in more-social wild carnivore species such as coatis (*Nasua nasua*) (77%)^63^, raccoons (*Procyon lotor*) (62%)^64^ and badgers (*Meles meles*) (57%)^26^.

In domestic cats, higher hemoplasma prevalence have been found in free-roaming domestic cats (e.g. 37% in Japan^20^) than in confined cats in urban areas (2–8% Valdivia city, Chile^43^; 4% different USA cities^18^), probably because greater exposure with bloodsucking arthropods and a higher incidence of wounds increase their exposure to hemoplasmas^18^. In our study, the proportion of infected cats ranged from 11% to 42%, depending on the area, and was not significantly associated with tick or fly infestation. We found no differences in hemoplasma spp. infection rates between sex and age classes, although other authors have reported higher hemoplasma prevalence in older domestic cat males^12^. This may be because the free-ranging behavior of all the sampled domestic cats, regardless of sex or age, may increase their exposure to a variety of bloodsucking vectors (e.g., mosquitoes, horseflies) and lead to increased social contact, including fighting. In addition, the possibility of vertical transmission and/or onward horizontal transmission could explain the lack of significant differences on infection among age classes.

In our survey, the most prevalent hemoplasma species, both in guigna and domestic cats was *C*Mhm, followed by *Mhf*. This is similar to results from previous studies of other felid populations^12,20,21,23,68^. *C*Mhm may infect guignas and cats more persistently and efficiently than other hemoplasma species, or perhaps is less virulent, allowing the coexistence between host and bacteria. Alternatively, these differences could be associated to different transmission methods among hemoplasma species.

Molecular characterization of 16S rRNA gene (1289 bp) was obtained for 11 of the 24 hemoplasma-positive guignas and 22 of 40 hemoplasma-positive domestic cats. The relatively low success rates of the assays was due to the quality of field samples, which were sometimes suboptimal due to degradation in field conditions (e.g, roadkills). Additionally, fluctuating pathogen loads on blood samples, where are correlated with the state of infection of the host, may also have affected the efficient amplification of long gene fragments (more than 1000 bp).

Phylogenetic analysis revealed well-supported clades that corresponded with the three identified hemoplasma species. NtSTs from sampled guignas and domestic cats from Chile were positioned within clades shared by domestic cats and wild felid and carnivore host species from around the world, suggesting a worldwide distribution of hemoplasmas and no phylogeographic differentiation among continents within each species-specific clade. Worldwide interspecific transmission of these pathogens between wild and domestic carnivore host species has been described by other authors^23,25,27^.

The ntST networks and genetic relationships among guigna and domestic cat hemoplasma ntSTs, *Mhf* and *Mycoplasma* sp. showed some evidence of cross-species transmission, as there was one shared ntST in each of these hemoplasma species. For *Mhf*, we also observed private ntSTs for domestic cats and for guignas (including that with the higher frequency in this species), and no genetic structure between hosts.

However, *C*Mhm ntST networks of sampled guignas and domestic cats from Chile showed a clear pattern of genetic clustering by host species, where all ntSTs were either unique to domestic cats or to guignas, and shared ntSTs were not found. The most prevalent ntSTs in guigna were all unique to this species. Therefore, a statistically significant high-level of genetic structure between guigna and domestic cat hemoplasma ntSTs was found. These results suggest that cross-species transmission events between both hosts may occur for some hemoplasma species but are most-likely relatively rare. As other authors have suggested^23,27^, domestic cats may be the source of the global distribution of multiple strains of hemoplasmas in wild felids, and these may persist through onward transmission following spillover. Overall, we found evidence for higher levels of intraspecific, versus interspecific, transmission in both guignas and domestic cats. This pattern is also supported by the analysis of landscape features, which found significant associations between higher hemoplasma prevalence of infection in guignas with pristine, continuous native forest devoid of domestic cats. Therefore, contrary to our hypothesis and the conceptual framework for pathogen emergence in wildlife, domestic cats in human-dominated landscapes appear not to be reservoirs or main drivers of hemoplasma infection in guignas.

Other authors have also described higher prevalence of hemoplasma infection in wild species inhabiting pristine areas compared to urban areas, including raccoons in USA^68^, where higher hemoplasma prevalence in pristine areas was associated with higher tick infestation rates in host species. In some cases, the clustered geographical distribution of hemoplasma, mainly in regions with warmer climates^69–72^, has been related to the role of an arthropod vector (mostly ticks) as one of the main transmission modes^29^. In our study, the widespread distribution of hemoplasma-positive animals in different seasons (cold and warm) and across study areas, including regions with marked differences in bioclimatic characteristics, and the higher prevalence of hemoplasma infected guignas in the rainy temperate bioclimatic area where ticks potentially associated to hemoplasma transmission are absent^73,74^, refute the idea that ticks are the main hemoplasma vectors. In this study, higher hemoplasma prevalence in pristine areas was not associated with higher tick or fly infestation rates in host species from these areas. Fleas were found in only 5.6% of evaluated domestic cats and no other ectoparasites were found infesting domestic cats or guignas. However, this does not rule out the possible role of other bloodsucking vectors, such as mosquitoes or horseflies.

On the other hand, guigna population densities are higher in pristine forest areas compared to fragmented landscapes^41^, suggesting hemoplasma transmission could be density-dependent. Therefore, direct transmission via infected blood or saliva^31,32^ should also be taken into account as a possible transmission mode explaining the widespread occurrence of hemoplasmas in wild guigna populations, especially considering possible transmission networks that include other forest-dwelling native carnivores (e.g., Darwin’s fox). No statistical differences on infection prevalence between adults and juveniles in guigna reveal the possibility of transplacental or vertical transmission, as well as horizontal onward transmission. The absence of statistical differences in infection prevalence among seasons suggests that there might be different transmission pathways, perhaps including one or more species of bloodsucking vectors (e.g. mosquitoes, horseflies), in addition to direct (vertical and horizontal) transmission.

Phylogenetic analysis also revealed the presence of a well-supported clade, not related to any previously described mycoplasma species worldwide. Two guignas and one domestic cat from Chile were positioned within this clade (shared ntST), which is also shared with a hemoplasma sequences found in Darwin’s fox from Chile and a Brazilian rodent. This previously undescribed *Mycoplasma* sp. group may correspond to a new species of hemoplasma, although this hypothesis must be further validated. Whether fox-guigna transmission occurs, or both individuals got infected through predation on rodents, is unknown. Interestingly, the Darwin’s fox is a forest-specialist carnivore, co-occurring with guignas in most of their distribution ranges, supporting the hypothesis that hemoplasma infection in guigna is associated to pristine, continuous native forest landscapes.

The pathogenicity of hemoplasma infection in wild felids is uncertain^75^, but most wild felids have been asymptomatic^22,25,26,76^. However, clinical signs (pale mucous membranes and nonregenerative anemia) associated with hemoplasma infection have been observed in some species, including the the Iberian lynx^23^. In our study, two hemoplasma infected guignas (juvenile males), of a total of 11 positive guignas for which hematological analysis were conducted, presented nonregenerative anemia. Both were infected by *Mhf* and one was coinfected with *C*Mhm. In domestic cats, *Mhf* has been described as the most pathogenic hemoplasma species^11–13,28^, which may explain our findings in guignas. However, *Mhf*-infected guignas not showing clinical signs were also found. The lack of differences in hemoplasma spp. infection prevalence between adult and juvenile guignas and the presence of infected animals in all seasons makes it difficult to define if these are endemic pathogens in guigna populations, or how long they persist in infected individuals. These infections may be chronic in some cases, as has been suggested by other authors in domestic cats^10^, showing mild or no clinical implications. Hence more health studies correlated with hematological analysis are necessary to clarify the pathogenic importance of hemoplasma infection in guignas.

In a related study, we observed high genetic similarity between domestic cat and guigna FIV and FeLV sequences and widespread shared ntSTs, suggesting that domestic cats were the source of infection^40^ (unpublished data). In contrast, our results here indicate that guigna infection patterns with mycoplasma species were not associated with human-modified landscapes, but instead, was significantly linked with pristine continuous forests. This indicates that modes of transmission of hemoplasma species and FeLV/FIV are different. Further effort is needed to document the ecological interactions among guigna and other forest-dwelling wild felids and carnivores (e.g. Darwin’s fox), which are currently unknown in southern Chile. Understanding multi-host pathogens, including hemoplasma species, which can be transmitted among numerous host species and via diverse transmission pathways, has critical implications for host species management strategies. Future studies should therefore focus on clarifying hemoplasma transmission patterns, characterizing the role of native carnivore species have in transmission networks, and documenting the pathogenic significance of feline hemoplasma infections in wild felid populations.

## Supplementary information

Assessing cross-species transmission of hemoplasmas at the wild-domestic felid interface in Chile using genetic and landscape variables analysis

## Data Availability

Derived data supporting the findings of this study are available from the corresponding author upon request.
