# Supplementary material for: Assessing cross-species transmission of hemoplasmas at the wild-domestic felid interface in Chile using genetic and landscape variables analysis
Source: Sci Rep. 2019 Nov 14;9:16816. doi: 10.1038/s41598-019-53184-4 (PMC6856521; doi:10.1038/s41598-019-53184-4)
Supplement: Supplementary file 1 — Assessing cross-species transmission of hemoplasmas at the wild-domestic felid interface in Chile using genetic and landscape variables analysis [file 41598_2019_53184_MOESM1_ESM.pdf]

Assessing cross-species transmission of hemoplasmas at the wild-domestic felid interface in Chile using genetic and landscape variables analysis

Sacristán I[1], Acuña F[2], Aguilar E[2], García S[2], López MJ[2], Cevidanes A[1], Cabello J[3,4], Hidalgo-Hermoso E[5], Johnson WE[6,7], Poulin E[8,9], Millán J[10], Napolitano C[8,11*]

SUPPLEMENTARY MATERIAL

Table S1. Best model representing multivariable relationships between predictor variables and total hemoplasma species, *Candidatus* Mycoplasma haemominutum and *Mycoplasma haemofelis* infection in guignas, using logistic regression analysis (n=102). Season variable was evaluated in 100 samples.

		**Regression coefficient**	**SE**	**Adjuste odds ratio**	**95% confidence interval**	***P* value**	**Hosmer Lemeshow test**
**Total hemoplasma spp**	Fragmented landscape	-2.50	0.94	0.08	-4.52-0.77	0.008	
	Chiloe Island	1.03	0.87	2.81	-0.67-2.84	0.23	
	Rainy temperate area	2.11	0.85	8.25	0.50-3.93	0.01	*p*-value=0.7
	Oceanic cold temperate area	0.70	1.33	2.03	-1.86-3.45	0.59	
	Warm season	-0.40	0.48	0.65	0.25-1.73	0.38	
***Candidatus* Mycoplasma haemominutum**	Fragmented landscape	-1.70	0.86	0.18	-3.47-0.05	0.04	*p*-value =0.6
	Chiloe Island	1.90	1.21	6.69	-0.23-4.97	0.12	
	Rainy temperate area	2.62	1.17	13.85	-3.47-0.05	0.02	
	Oceanic cold temperate area	1.19	1.51	3.315	0.49-4.36	0.43	
	Warm season	-0.16	0.57	0.85	0.28-2.72	0.77	
Mycoplasma haemofelis	Fragmented landscape	-2.48	1.10	0.08	-4.87 -0.43	0.02	*p*-value =0.6
	Chiloe Island	-0.18	1.21	0.83	-2.74- 2.23	0.88	
	Rainy temperate area	0.06	1.19	1.06	-2.50- 2.44	0.96	
	Ocenaic cold temperate area	0.64	1.50	1.09	-2.34-3.73	0.67	
	Warm season	-0.29	0.68	0.76	0.20-3.18	0.67	

Table S2. Best model representing multivariable relationships between predictor variables and total hemoplasma species, *Candidatus* Mycoplasma haemominutum and *Mycoplasma haemofelis* infection in domestic cats, using logistic regression analysis (n=262). Season variable was evaluated in 213 samples.

		Regression coefficient	SE	Adjuste odds ratio	95% confidence interval	*P* value	Hosmer Lemeshow test
**Total hemoplasma spp.**	Sex male	0.71	0.36	2.04	1.02-4.26	0.05	
	Age juvenile	-0.82	0.65	0.44	0.09-1.35	0.20	*p*-value=0.11
	Mediterranean area	-0.57	0.56	0.6	0.17-1.6	0.30	
	Rainy temperate area	-0.70	0.44	0.50	0.20-1.16	0.11	
	Oceanic-cold temperate área	-0.6	0.49	0.63	0.23-1.62	0.24	
	Warm season	-0.13	0.40	0.88	0.38-1.94	0.75	*p*-value=1.00
***Candidatus* Mycoplasma haemominutum**	Sex male	0.75	0.43	2.12	0.92-5.18	0.08	
	Age juvenile	-0.96	0.78	0.38	0.058-1.43	0.21	
	Mediterranean area	0.52	0. 64	1.68	0.46-5.98	0.41	*p*-value=0.15
	Rainy temperate area	-0.13	0.58	0.88	0.27-2.87	0.82	
	Oceanic-cold temperate área	0.65	0.58	1.90	0.61-6.27	0.26	
	Warm season	-0.15	0.43	0.85	0.35-2.0	0.72	*p*-value=1.00
Mycoplasma haemofelis	Sex male	0.45	0.55	1.59	0.55- 4.92	0.39	
	Age juvenile	-0.65	1.07	0.52	0.02- 2.90	0.54	
	Mediterranean area	-1.48	1.01	0.23	0.02-1.23	0.14	*p*-value=0.68
	Rainy temperate area	-0.79	0.60	0.45	0.13-1.42	0.18	
	Oceanic-cold temperate área	-2.02	1.09	0.13	0.01- 0.78	0.06	
	Warm season	0.02	0.72	1.01	0.23-4.5	0.97	*p*-value=1.00

Table S3. Hematological parameters of each hemoplasma positive guignas. Hematological normal values of *Leopardus geoffroyi*, the most closely genetically related species with the guigna, are provided for comparison.

**ID**	**Sex**	**Age**	**Hemoplasma species**	**Hemoplasma blood smear status**	**RBC ($x10^6/\mu L$)**	**Haemoglobin (g/dL)**	**Haematocrit (%)**	**MCV fL**	**MCHC g/dL**	**WBC ($x10^3/ \mu L^{-1}$)**	**Segmented neutrophil ($x10^3/ \mu L^{-1}$)**	**Lymphocyte ($x10^3/ \mu L^{-1}$)**	**Monocyte ($x10^3/ \mu L^{-1}$)**	**Eosinophil ($x10^3/ \mu L^{-1}$)**	**Platelets ($x10^5/ \mu L^{-1}$)**
Leopardus geoffroyi*	_-	_-	-_	-_	**6.71-9.25**	**11.5-14.9**	**35.2-47.8**	**47-55.8**	**30.2-35.6**	**5.38-14.22**	**3.35-9.16**	**1.03-3.15**	**0.06-0.56**	**0-1.44**	**2.80-3.86**
LG151	Male	Juvenile	*Mhf*	(-)	6.46 **	11.9	38.4	59.4**	31.0	3.80**	1.97**	1.63	0.19	0.00	4.15**
LG158	Female	Adult	*C*Mhm	(-)	8.74	15.9**	40.0	46.0	39.8**	10.60	8.48	1.06	0.10	0.00	4.45**
LG159	Male	Juvenile	*C*Mhm	(+)	6.67	12.0	38.0	57.0**	32.0	10.20	6.42	2.95	0.10	0.61	5.46**
LG160	Male	Adult	*C*Mhm+*Mhf*	(+)	6.73	12.8	39.0	57.0**	33.0	7.00	5.46	1.26	0.14	0.14	3.06
LG172	Male	Juvenile	*C*Mhm+*Mhf*	(-)	6.48**	13.3	35.0**	54.0	34.0	13.20	10.56**	1.71	0.66	0.00	1.66
LG175	Male	Juvenile	*C*Mhm + *Mhf*	(-)	6.92	13.8	44.9	65.0**	30.6	10.24	7.07	3.12	0.06	0.00	7.98**
LG176	Male	Adult	*Mhf*	(-)	7.32	13.6	44.1	60.0**	30.8	14.71	10.80**	3.06	0.86**	0.00	6.68**
LG177	Female	Adult	*Myco.sp.+Mhf*	(-)	8.89	15.9**	54.8**	62.0**	28.9**	21.70	10.24**	11.35**	0.11	0.00	6.64**
LG190	Male	Adult	*C*Mhm	(-)	9.31**	15.5**	48.4	52.0	32.0	5.20**	4.05	0.83**	0.26	0.05	1.96
LG191	Male	Adult	*C*Mhm	(-)	8.70	13.5	42.1	48.0	32.0	6.40	4.22	1.92	0.19	0.06	0.76
LG192	Female	Adult	*Mhf*	(-)	8.34	15.5**	46.9	56.0**	33.0	12.80	10.75**	1.40	0.51	0.12	1.92

*Hematological normal values of *Leopardus geoffroyi* [66]

**Abnormal hematological values in relation to the normal values of *Leopardus geoffroyi*.

Table S4. Serum biochemistry parameters of each hemoplasma positive guignas. Biochemical normal values of *Leopardus geoffroyi*, the most closely genetically related species with the guignas, are provided for comparison.

ID	Sex	Age	Hemoplasma species	Hemoplasma blood smear status	TP g/dL	Albumin g/dL	Globulin g/dL	TBIL mg/dL	ALT IU/L	FA IU/L	GGT IU/L	AST IU/L	Calcium mg/dL	Phosphorus mg/dL	Creatinine mg/dL	BUN mg/dL	Total cholesterol mg/dL	Glucose mg/dL
Leopardus geoffroyi*	**-**	**-**	**-_**	**-_**	**6.9-8.7**	**2.4-3.8**	**3.8-5.2**	**0.1-1.1**	**13-60**	**-**	**-**	**24-68**	**8.7-11.1**	**3.7-6.5**	**0.8-2.6**	**19-68**	**-**	**145-265**
LG158	Female	Adult	*C*Mhm	(-)	-	-	-	-	182**	-	-	258**	1.84	1.6	29.0**	14.4	-	-
LG159	Male	Juvenile	*C*Mhm	(+)	8.7	4.5**	4.2	0.20	69.0**	90	2.0	150**	8.6	6.0	1.6	49.0	212	82**
LG160	Male	Adult	*C*Mhm+*Mhf*	(+)	6.5	4.1**	2.4	0.40	77.0**	35	2.0	296**	9.5	5.9	1.2	50.0	188	107**
LG172	Male	Juvenile	*C*Mhm+*Mhf*	(-)	7.2	4.3**	2.9	0.20	56.8	35	3.2	152**	8.7	4.2	0.8	33.3	160	87**
LG175	Male	Juvenile	*C*Mhm + *Mhf*	(-)	11.2**	4.4**	6.8**	0.30	6.4	8000	2.9	176**	10.0	13.6**	0.4**	37.4	156	14**
LG176	Male	Adult	*Mhf*	(-)	8.4	3.8	4.6	0.24	47.0	40	4.3	162**	9.4	5.2	0.8	61.3	158	42**
LG177	Female	Adult	*Myco*.sp.+*Mhf*	(-)	9.6**	4.0**	5.6	0.37	32.4	50	8.2	134**	10.0	5.6	0.8	49.1	146	42**
LG190	Male	Adult	*C*Mhm	(-)	8.8**	3.3	5.7	0.26	76.3**	36.2	2.8	255**	8.6	3.3	1.0	41.0	226	103
LG191	Male	Adult	*C*Mhm	(-)	7.6	3.0	4.6	0.25	73.2**	42.9	2.5	261**	10.5	7.2	1.0	57.4	257	156
LG192	Female	Adult	*Mhf*	(-)	7.7	3.1	4.6	0.22	85.2**	35.1	1.0	195**	9.5	4.9	1.0	33.6	208	92**

*Biochemical normal values of *Leopardus geoffroyi*[66].

**Abnormal biochemical values in relation to the normal values of *Leopardus geoffroyi*.

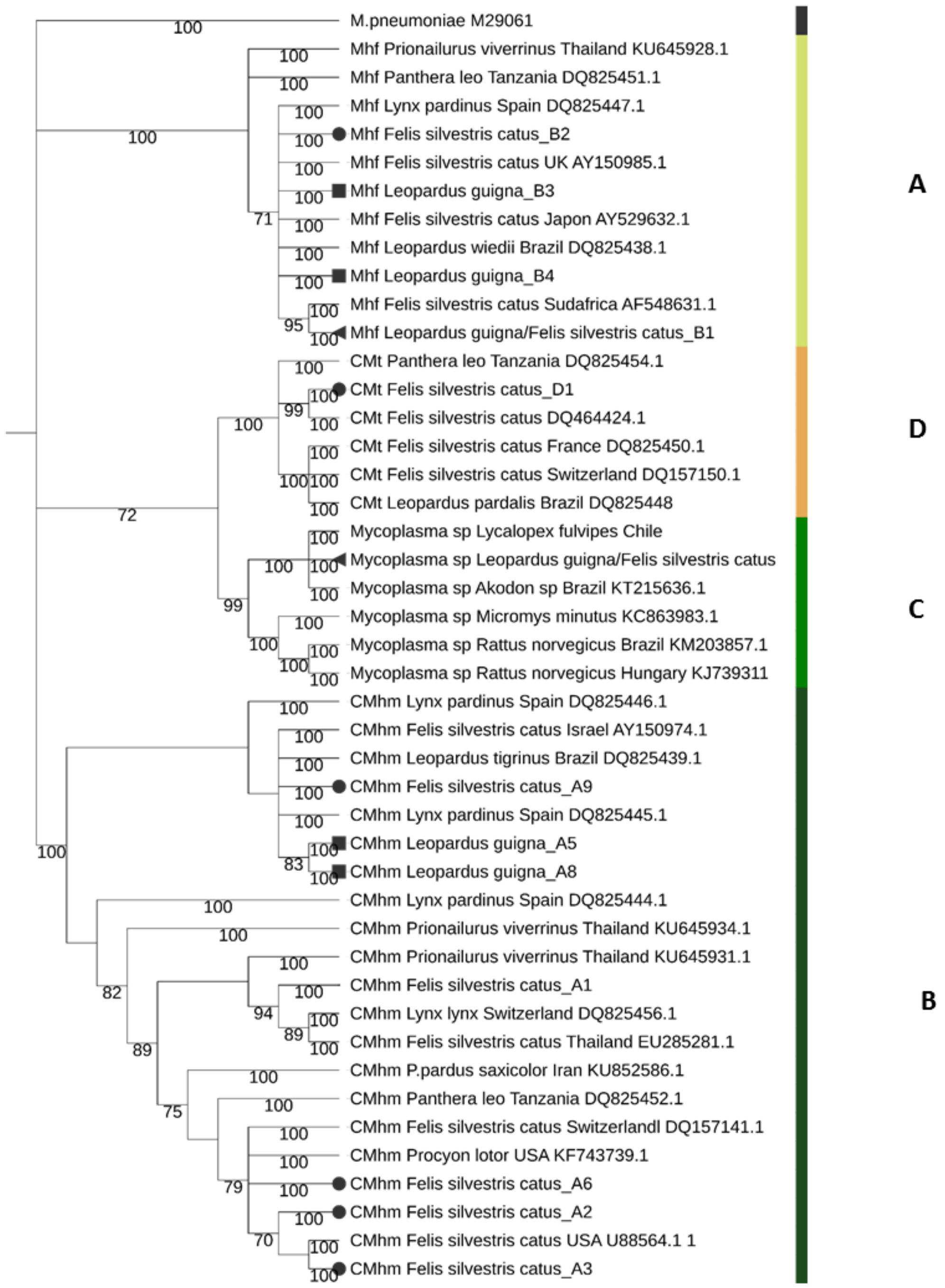

Figure S1. Bayesian tree of 944 bp of 16S rRNA gene for guigna and domestic cat sequences. *M. pneumniae* sequence has been used as outgroup. Posterior probabilities are given at the nodes of the tree, expressed as percentage, only values of ≥ 70 are shown. Circles, squares and diamonds show domestic cat ntST, guigna ntST and shared ntST from this study.

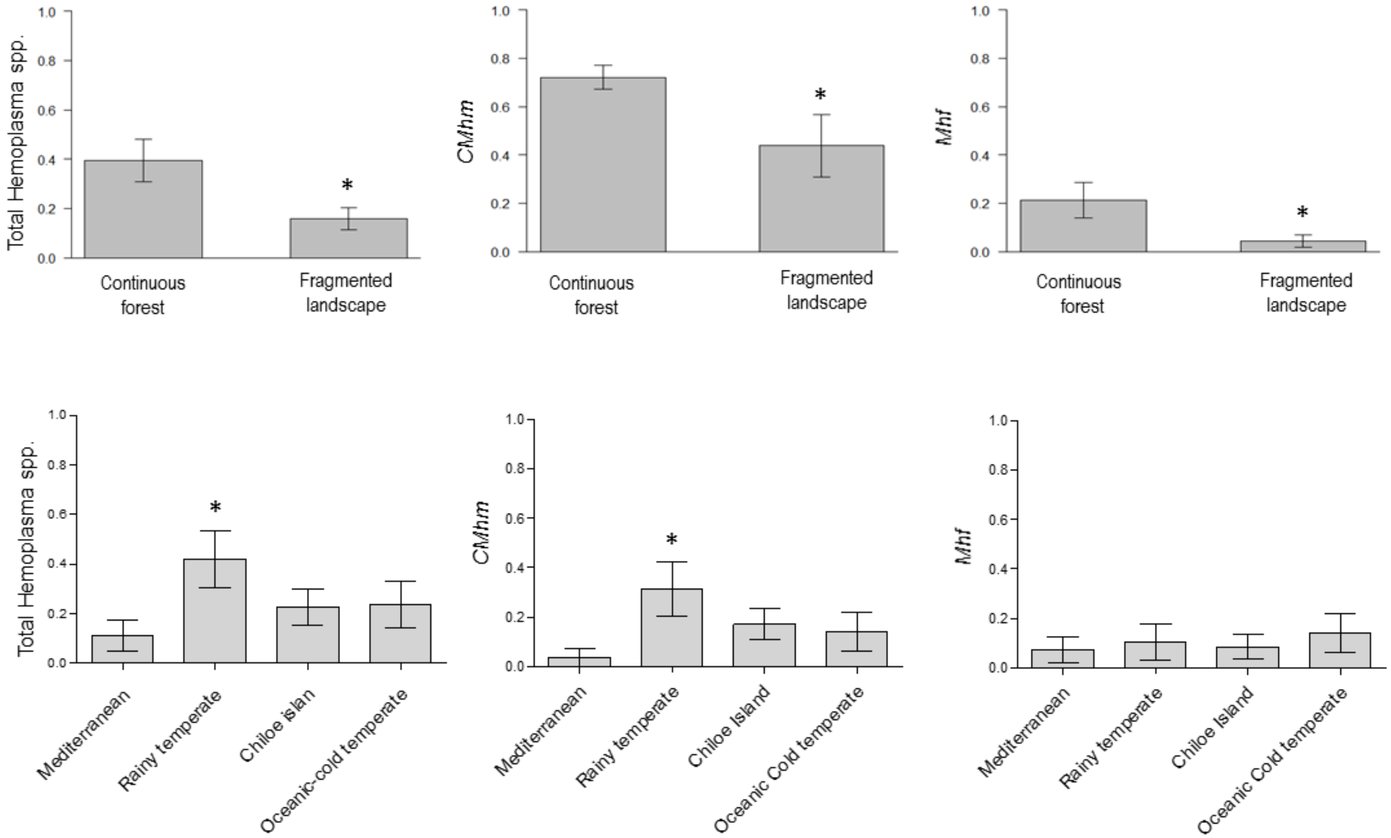

Figure S2. Bar graphs representing the prevalence of total hemoplasma species the two more prevalent hemoplasma species (*Candidatus* Mycoplasma haemominutum and *Mycopalasma haemofelis)* depending on the different bioclimatic areas of study and land use variables.

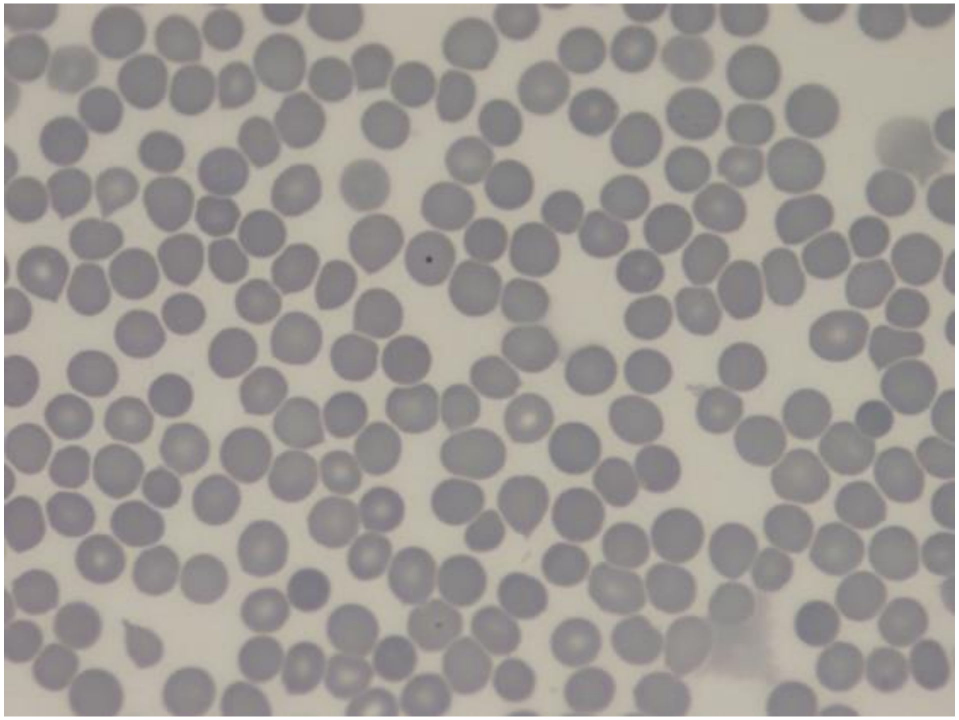

Figure S3. Blood smear images showing a structure compatible with hemoplasma.

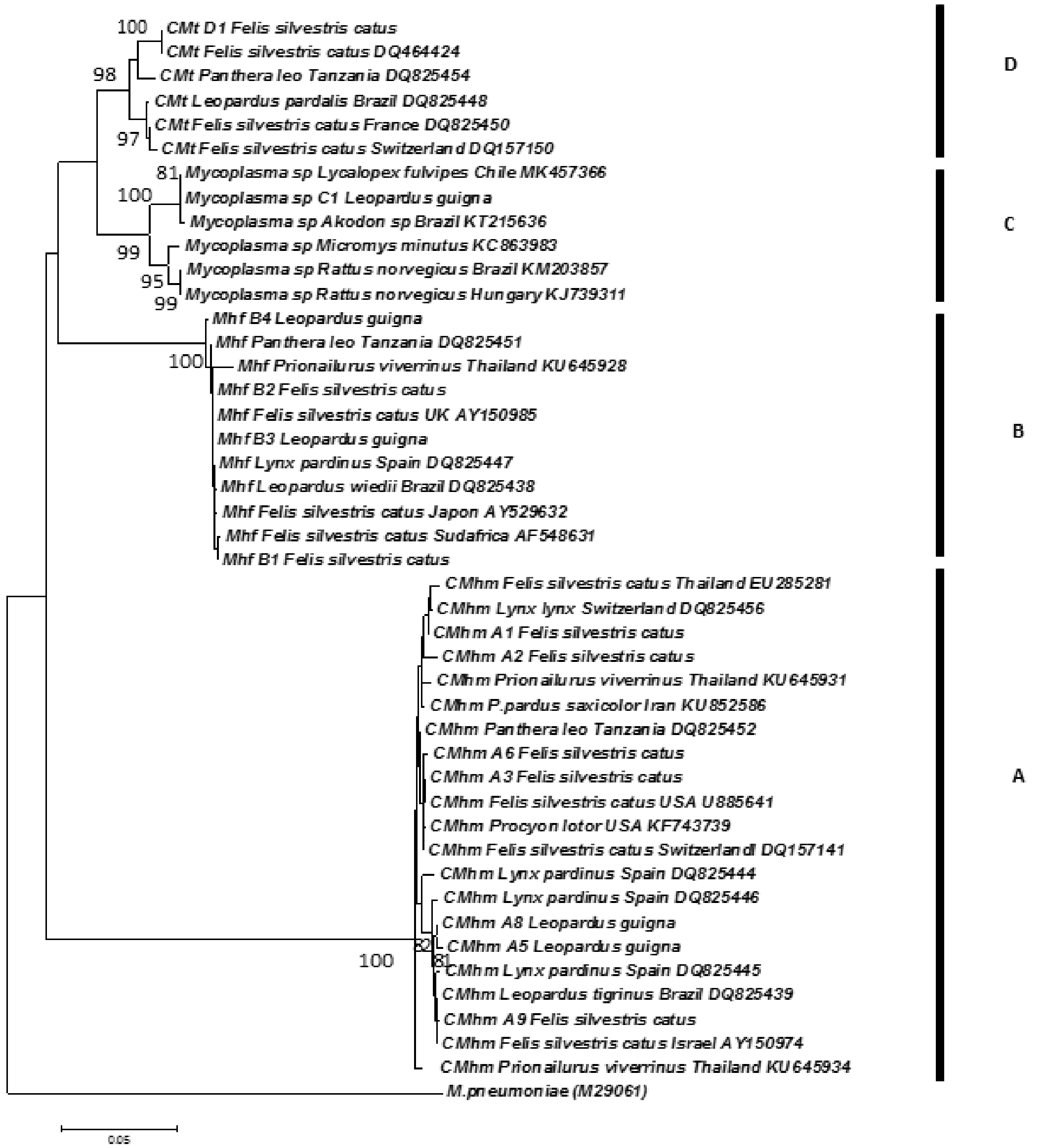

Figure S4. Neighbor-joining tree of 944 bp of the 16S rRNA gene for guigna and domestic cat. *M. pneumniae* sequence has been used as outgroup. Bootstrap values of ≥ 70 are printed at the nodes of the tree. The Bayesian phylogenetic tree and maximum likelihood phylogenetic tree were congruent. The four phylogenetic (taxonomic) groups are labelled A, B, C and D.
